# Selective Feeding of Bdelloid Rotifers in River Biofilms

**DOI:** 10.1371/journal.pone.0075352

**Published:** 2013-09-20

**Authors:** Benoit Mialet, Nabil Majdi, Micky Tackx, Frédéric Azémar, Evelyne Buffan-Dubau

**Affiliations:** 1 Université de Toulouse, INP, UPS, Ecolab (Laboratoire d’Ecologie Fonctionnelle et Environnement), Toulouse, France; 2 Centre National de la Recherche Scientifique, Ecolab, Toulouse, France; 3 Université de la Rochelle, CNRS, UMR 7266 LIENSs (Littoral Environnement et Sociétés), La Rochelle, France; University of Connecticut, United States of America

## Abstract

*In situ* pigment contents of biofilm-dwelling bdelloid rotifers of the Garonne River (France) were measured by high performance liquid chromatography (HPLC) and compared with pigment composition of surrounding biofilm microphytobenthic communities. Among pigments that were detected in rotifers, the presence of carotenoids fucoxanthin and myxoxanthophyll showed that the rotifers fed on diatoms and cyanobacteria. Unexpectedly, while diatoms strongly dominated microphytobenthic communities in terms of biomass, HPLC results hinted that rotifers selectively ingested benthic filamentous cyanobacteria. In doing so, rotifers could daily remove a substantial fraction (up to 28%) of this cyanobacterial biomass. The possibility that the rotifers hosted symbiotic myxoxanthophyll-containing cyanobacteria was examined by localisation of chlorophyll fluorescence within rotifers using confocal laser scanning microscopy (CLSM). CLSM results showed an even distribution of quasi–circular fluorescent objects (FO) throughout rotifer bodies, whereas myxoxanthophyll is a biomarker pigment of filamentous cyanobacteria, so the hypothesis was rejected. Our results also suggest that rotifers converted β-carotene (provided by ingested algae) into echinenone, a photoprotective pigment. This study, which is the first one to detail *in situ* pigment contents of rotifers, clearly shows that the role of cyanobacteria as a food source for meiobenthic invertebrates has been underestimated so far, and deserves urgent consideration.

## Introduction

The epilithic biofilm is a complex assemblage of organisms (bacteria, fungi, algae, heterotrophic protozoans and metazoans) embedded in a mucous matrix of exopolymeric substances (EPS) together with entrapped organic and inorganic particles [1,2]. This mat coats any hard submerged substrate, and when enough light is available, microphytes (and their EPS exudates) contribute copiously to its organic content, then fuelling biofilm-dwelling consumers [1,3]. In the Garonne River, this biofilm is deeply impacted by hydrological scenarios, and consequently the development of its fauna and flora depends on its stability [4-6]. Even if diatoms usually dominate the biofilm phototrophic community in terms of biomass, fluctuating contributions of green algae and cyanobacteria can occur [7,8], constraining resource availability for biofilm-dwelling grazers.

Meiobenthic organisms (meiofauna) are, by definition, benthic invertebrates passing through 500 µm meshes but retained on 42 µm meshes [9]. Through its activity (e.g. bioturbation, grazing and excretion), the meiofauna can affect key processes such as primary production, bacterial development and organic matter fluxes [10-15]. A few recent studies address biofilm-dwelling meiofauna feeding habits, highlighting the importance of pelagic to benthic vertical imports through rotifer and ciliate filtration activity [16], or exploring nematode feeding selectivity to budget their implication in phototrophic carbon transfer [17,18].

Hence, understanding meiofauna trophic role is pivotal to disentangle energy flows in many freshwater food webs [19-21], given that their high production to biomass ratios places meiofauna as relevant trophic intermediaries between micro- and macro-organisms [20-22]. However, this issue, and especially the quantitative and qualitative *in situ* uptake of primary producers by meiofauna still receives little consideration in freshwater systems [10,17,18,22-25].

Rotifers are important contributors to freshwater meiofauna and constitute significant prey for macrobenthic and other meiobenthic invertebrates [26,27]. In the Garonne River, bdelloid rotifers frequently dwell epilithic biofilms [6]. Bdelloid rotifers can consume a wide variety of preys (algae, bacteria and yeasts) either by suspension feeding, scraping or browsing [28]. While some rotifer species are known to feed selectively [26], the feeding behaviour of Bdelloids is poorly documented and *in situ* data are particularly lacking. For biofilm-dwelling rotifers, this is mostly due to the difficulty of measuring their feeding behaviour in such complex habitats: not only are epilithic biofilms composed of a complex matrix containing a variety of potential food sources, but the mucous nature of the biofilm itself poses practical experimental problems.

High-performance liquid chromatography (HPLC) quantification of biomarker pigments contained in guts is useful to obtain quantitative and qualitative *in situ* data on the diet of post-mortem isolated taxa of animals on characterised preys (e.g. microphytic groups). To date, this technique is routinely used to detail the feeding behaviour of aquatic invertebrates in planktonic and benthic habitats (e.g. [29,30]). Especially, HPLC gut pigment quantification allows to examine selective microphyte feeding within complex-clustered benthic habitats such as muds or biofilms [18,31].

As stated above, the epilithic biofilm can offer a wide range of potential substrates for the growth of its inhabiting rotifer community. The aim of this study was: (1) to quantify *in situ* meiobenthic rotifer feeding activity and possible selectivity; (2) to calculate the pressure exerted on the various potential microphytic prey groups as identified by marker pigments, to evaluate the importance of these for the biofilm phototrophic biomass dynamic.

## Material and Methods

### Study site and sampling procedures

In the middle course of the Garonne River, the river bed displays mainly cobble bar clusters. During low-water periods, a thick biofilm typically coats the upper surface of cobbles (Figure S1). One of these cobble bars was sampled (01°17’53″ E, 43°23’45″ N; elevation 175 m a.s.l.) at three occasions (19/11/2008, 01/04/2009 and 15/03/2010), where biofilm-dwelling rotifers were particularly abundant [6]. During sampling occasions, water depth ranged from 30 to 50 cm, flow velocity ranged from 0.02 to 0.62 m s^-1^ and water temperature ranged from 5.3 to 9.2 °C.

Twelve biofilm covered cobbles (diameter: ~10 cm) were hand-collected at each sampling occasion: (1) 4 cobbles to determine rotifer density and biomass, (2) 4 cobbles to measure biofilm ash-free dry mass (AFDM), (3) 4 cobbles to measure biofilm microphytobenthos (MPB) pigment concentrations using HPLC-analysis and to estimate the relative contribution of the different MPB groups to total MPB biomass in terms of chlorophyll *a* (Chl *a*) using CHEMTAX version 1.95 software [32]. Biofilm samples were gathered by scraping the upper surface of the cobbles with a scalpel and a toothbrush. Scraped cobbles were photographed and the area of the surface from which biofilm had been removed (clearly visible on the cobble) was measured (ImageJ software, version 1.38 [33]), to obtain data per area unit. Further processing of these samples to obtain rotifer densities, biofilm AFDM, pigment quantifications by HPLC and MPB community composition was detailed in Majdi et al. [6,8].

For rotifer pigments analyses, three additional cobbles were collected on each sampling occasion. Biofilm was collected as described above and immediately preserved within liquid N_2_. Instant freezing minimizes gut content egestion [34]. These frozen samples were then stored at -80°C until rotifer sorting for gut pigment analyses.

### Rotifer sorting for pigment analyses

A frozen biofilm sample was thawed, then suspended in 2 L tap water. A water jet was used to mix the biofilm suspension in order to facilitate the separation of particles heavier than rotifers by decantation following an elutriation procedure [35]. After 2 minutes of decantation, the supernatant was poured through a 40 µm sieve to retain rotifers. A large majority of the rotifers found in our samples belonged to the Bdelloidea phylum (80–95% of their abundance). Thus, we selected only bdelloid individuals. Small groups of 10–40 undamaged bdelloids were sorted under a stereomicroscope (x 60 magnification) using a needle picker. Each group of rotifers was thoroughly transferred with a 10 µL pipette to a cold ultrapure water bath (MilliQ filtration; Millipore, Billerica, MA, USA) to rinse body-adhering particles. For each sample, 418 to 946 bdelloids were isolated, photographed by groups of 40 individuals, and then placed in an Eppendorf tube containing 500 µL ultrapure water. Three replicate samples were treated for each sampling occasion, leading to a total of 4408 sorted bdelloid individuals. Note that bdelloid rotifers were sorted under minimum light exposure and above a thin ice block to limit pigment photo- and thermo-degradation.

### Extraction of rotifer pigments

Each sample of sorted bdelloids was centrifuged (500 g, 5 min) to allow the settlement of a “bdelloid pellet”. Excess water was removed by freeze-drying, then pigments were extracted from rotifer samples in 200 µL of 98% cold-buffered methanol (with 2% of 1 M ammonium acetate) by sonicating for 90 seconds in an ultrasonic bath (Elmasonic S-10 series, IMLAB, Lille, France). Extraction was then allowed for 15 min at -20°C in the dark. The pigment extract so obtained was then filtered (0.2 µm PTFE syringe filter with very low dead volume <10 µL, ReZist series Ø13 mm, Whatman inc., Florham Park, NJ, USA) and immediately analyzed using high performance liquid chromatography (HPLC).

#### HPLC-analysis of rotifer pigment samples

HPLC analyses of rotifer pigment samples were performed using a liquid chromatograph consisting of a 100 µL loop auto-sampler and a quaternary solvent delivery system coupled to a diode array spectrophotometer (LC1200 series, Agilent technologies inc., Santa Clara, CA, USA). The mobile phase was prepared and programmed according to the analytical gradient protocol described in Barlow et al [36]. Pigment separation was performed through a C8, 5 µm column (MOS-2 HYPERSIL, Thermo Fisher scientific inc., Waltham, MA, USA). The diode array detector was set at 440 nm to detect carotenoids, at 665 nm for chlorophylls and pheopigments [37]. Data analysis was performed using ChemStation software (version A.10.02, Agilent technologies inc.). Pigments were identified by comparing their retention time and absorption spectra with those of pure standard pigments (DHI LAB products, Hørsholm, Denmark). Each pigment concentration was calculated by relating the peak area of its chromatogram with the corresponding area of calibrated standard. Fucoxanthin and chlorophyll *a* pigments that were spectrally similar but did not have the same retention time as standards were designated “like-pigments”, and were quantified using the response factor obtained from standards.

### Rotifer dry mass and ingestion rates

For each rotifer sample, 100 individuals to be isolated for gut pigment measurement were randomly measured, with ImageJ software. Rotifer individual wet mass (WM), was calculated from biometric conversion of their body dimensions and dry mass converted from WM, as follows [9] : WM = 0.26LW^2^×1.028; DM = 0.1WM.

To calculate ingestion rates of organisms from their gut contents, gut passage times (GPT) are required. Such data are very rare for meiobenthic rotifers. Only one study provides an indication about the gut passage time (GPT) of two species of sessile benthic rotifers: > 30 min [38]. However, some values of GPT are given for planktonic rotifers showing that it is variable among species (from 16 to 50 min) and also for one species under experimental conditions (from 30 to 45 min) [38]. In this context, we considered a range of ingestion rates based on GPT values spanning 16 to 50 min. Since Chl *a* is converted into pheopigment during digestive processes (e.g. [29]), we considered the Chl *a*-equivalent (Chl *a*-eq), which is the sum of Chl *a* with its degradation products: pheophorbide *a* and pheophytin *a*, as a proxy representing the total phototrophic biomass ingested by bdelloids. Individual ingestion rates (µg Chl *a*-eq ind.^-1^ day^-1^) were multiplied by the rotifer densities to obtain ingestion rates per surface unit. Ingestion rates were expressed as a percentage of the microphytopigments standing stock at the day of sampling, to estimate the grazing pressure of the rotifer community (% day^-1^) on MPB biomass.

#### Pigment localisation

Since pigments originated from endosymbionts have been observed in other benthic organisms such as neotropical snails or mesophotic temperate sponges [39,40], we envisaged that pigment detection in rotifers could potentially also reveal the existence of endosymbionts. Thus, we also looked at the distribution of the pigmented items within the body of the rotifers using confocal laser microscopy. We specifically verified the form and the positioning of the fluorescent objects within the bdelloid bodies in order to see if these corresponded to the ingested algae or were positioned close to the body border, as would be expected from phytobionts.

Five bdelloid rotifers sorted from freshly thawed biofilm samples were mounted on slides and photographed under a Leica TCS SP2 confocal laser scanning microscope (CLSM), with 488-nm laser emission and 665-nm fluorescence record (i.e. typical Chl *a* fluorescence signature). Rotifer axial profiles were scanned with a slice step of 2 µm. Using ImageJ software, we measured the area, corrected total cell fluorescence (CTCF) and circularity of fluorescent objects (FO) detected on the centremost slice. FO were also localised regarding their distance to the nearest rotifer body border. This distance was corrected regarding rotifer maximum radius. The frequency distribution of FO was fitted using χ^2^ test. The correlations between FO characteristics and their relative distance to the nearest rotifer body border were explored through multiple linear correlations. These tests were performed using STATISTICA software (version 8.0, Statsoft inc., Tulsa, OK, USA).

## Results

**Figure 1:**
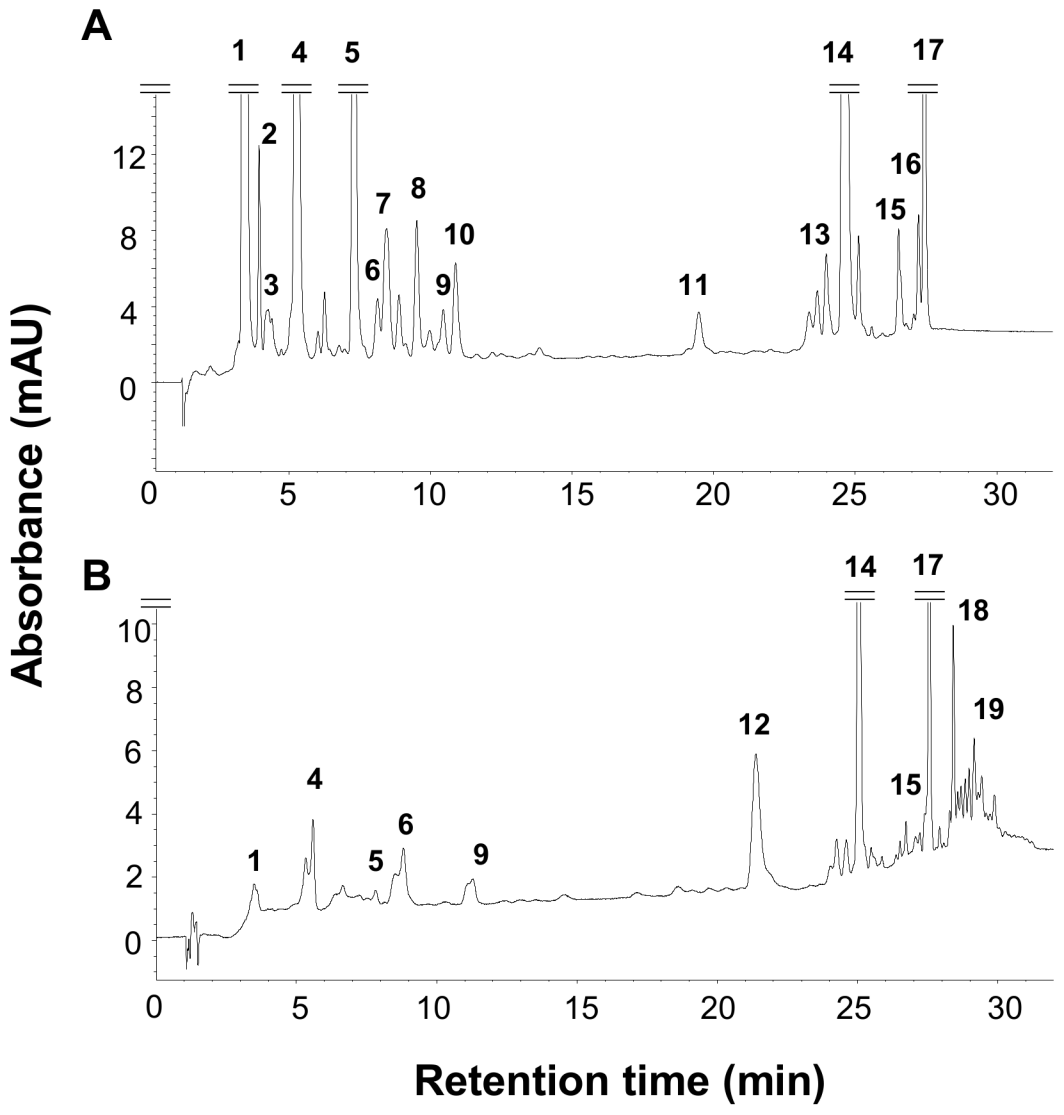
Examples of HPLC absorbance chromatograms obtained at 440 nm from field samples. (A) biofilm, (B): rotifer (946 individuals). 1: chlorophylls *c*; 2: fucoxanthin-like; 3: pheophorbide *a*; 4: fucoxanthin; 5: diadinoxanthin; 6: myxoxanthophyll; 7: cis-fucoxanthin; 8: zeaxanthin-like; 9: zeaxanthin; 10: lutein; 11: chlorophyll b; 12: echinenone; 13: chlorophyll a-like; 14: chlorophyll *a*; 15: pheophytin *a*; 16: α-carotene; 17: β-carotene 18: echinenone-like1; 19: echinenone-like 2.

Biomarker pigments that were detected in biofilm samples were (a) chlorophyll *c*, fucoxanthin and diadinoxanthin, (b) lutein and chlorophyll b and (c) zeaxanthin and myxoxanthophyll, indicating the presence of diatoms (a), green microalgae (b) and cyanobacteria (c) respectively (Table 1, Figure 1 a). Myxoxanthophyll is a characteristic carotenoid of some filamentous cyanobacteria whereas zeaxanthin can also originate from some green algae [41,42]. In terms of biomass, diatoms strongly dominated biofilm phototrophic community (Table 2).

**Table 1:** Range of concentrations for the major pigments detected in field samples.

**No.**	**Pigments**	**Biofilm**	**Rotifers**	**Prob. Source**	**Ref.**
		**(µg cm^-2^**)	**(µg mgDW^-1^**)		
1	Chlorophyll *c*	1.00–10.10	0.11-0.64	D	[71]
2	Fucoxanthin-like	*	ND		
3	Pheophorbide *a*	0.06–0.38	0.00-0.05	All	[71]
4	Fucoxanthin	3.60–42.00	0.06-0.88	D	[71]
5	Diadinoxanthin	0.50–7.70	0.03-0.20	D	[71]
6	Myxoxanthophyll	0.02–0.32	0.19-0.98	FC	[41,42]
7	Cis-fucoxanthin	*	ND		
8	Zeaxanthin-like	*	ND		
9	Zeaxanthin	0.08–0.30	0.06-0.240	C, GA	[71]
10	Lutein	0.11–0.39	ND	GA	[71]
11	Chlorophyll b	0.14–0.59	ND	GA	[71]
12	Echinenone	ND	0.53-0.72	C/Inv	[43,67]
13	Chlorophyll a-like	*	*		
14	Chlorophyll a †	15.50–81.10	2.01-5.40	All	[71]
15	Pheophytin a	0.24–1.70	0.46-1.04	All	[71]
16	α-carotene	*	ND		
17	β–Carotene	1.00–1.40	0.34-0.77	All	[71]
18	Echinenone-like 1	ND	*		
19	Echinenone-like 2	ND	*		

Pigments are listed following their elution order. Literature references used to determine the likely algal sources: Used abbreviations: D: diatoms; All: all microalgal taxa; FC: filamentous cyanobacteria; C: cyanobacteria; GA: green microalgae; Inv: invertebrates. * detected but not quantified; ND: = not detected. † Chlorophyll a quantification = chlorophyll *a* + chlorophyll *a* like.

Echinenone and Myxoxanthophyll, both characteristic of cyanobacteria, were present in relative high concentration in bdelloid rotifers (Table 1, Figure 1b). Myxoxanthophyll has not been reported as an animal carotenoid [43], and is characteristic of filamentous cyanobacterial taxa [41,42]. Hence, its presence among rotifer pigments reveals that they fed on biofilm filamentous cyanobacteria. The presence of chlorophylls *c*, fucoxanthin and diadinoxanthin among rotifer pigments indicates that they also fed on diatoms (Table 1, Figure 1b). Lutein and chlorophyll b were not detected in rotifers suggesting that they did not feed on green microalgae.

**Table 2:** Algal and rotifer biomasses in the biofilm samples.

		**Nov. 2008**	**Apr. 2009**	**Mar. 2010**
**Biofilm**				
Biomass (AFDM)	mg cm^-2^	2.8 ± 0.5	5.5 ± 0.8	15.7± 5.1
Chlorophyll a	µg cm^-²^	15.5 ± 3	40 ± 5.5	81 ± 27
Diatoms	%	76.4	94.2	98.2
Green algae	%	19.6	5.5	1.8
Cyanobacteria	%	4.0	0.3	traces
**Rotifers in biofilm**				
Individual mean dry weight	ng	35.0	45.3	34.6
Density	ind cm^-2^	35.3 ± 35.9	126.3 ± 24.4	83.3 ± 11.7

Total biofilm biomass (ash free dry mass: AFDM), microphytobenthic biomass (in term of Chl *a*), density and biomass of biofilm-associated rotifers (mean values ± SD, n = 4) are given. Relative biomasses of algal groups are assessed by CHEMTAX and given in % of total microphytic biomass (in term of Chl *a*). ND: no data.

**Figure 2:**
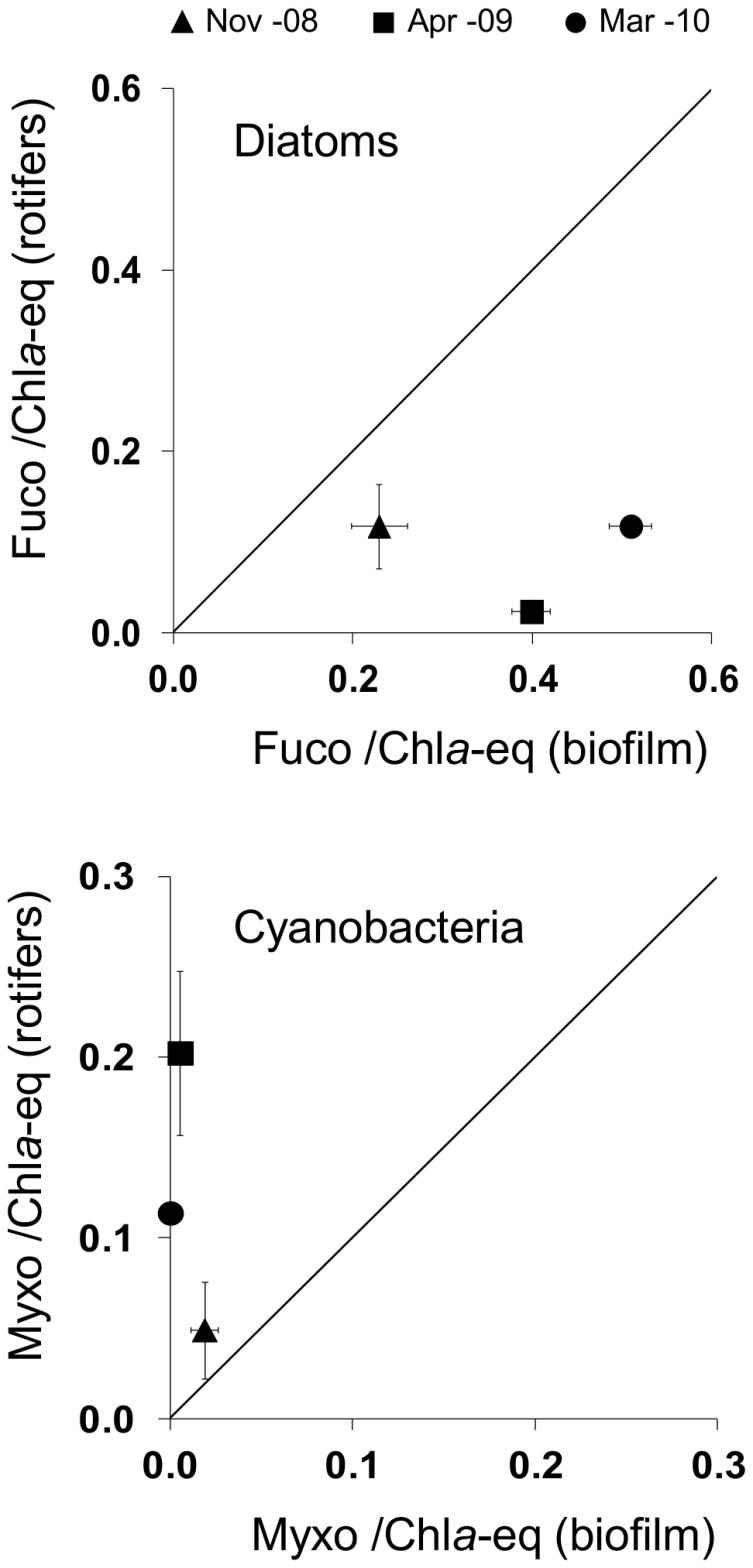
Comparison of pigment proportions between rotifer gut contents (rotifers) and their habitat (biofilm). (A) Fucoxanthin: Chl*a*-eq; (B): Myxoxanthophyll:Chl*a*-eq; Error bars are SD (n = 3). Fucoxanthin (Fuco) and myxoxanthophyll (myxo) are biomarkers for diatoms and filamentous cyanobacteria respectively.

Bdelloid fucoxanthin/Chl *a*-eq and myxoxanthophyll/Chl *a*-eq ratios (i.e. respective biomass proportions of diatoms and cyanobacteria to total microphytes in the rotifer gut) were compared to fucoxanthin/Chl *a*-eq and myxoxanthophyll/Chl *a*-eq ratios in the biofilm to detect any potential selective uptake of diatoms and/or cyanobacteria. Results are displayed in Figure 2: Rotifer fucoxanthin/Chl *a*-eq was lower than biofilm fucoxanthin/Chl *a*, showing that diatoms were ingested in lower proportions than their availability in the biofilm. In contrast, rotifer myxoxanthophyll/Chl *a*-eq was much higher than biofilm myxoxanthophyll/Chl *a*. This shows that bdelloids consumed cyanobacteria in much higher proportions than their availability within the biofilm matrix. Ingestion rates and the maximum feeding pressure exerted by bdelloid rotifers on various potential sources (total phototrophic biomass, cyanobacteria, diatoms) were estimated assuming that the rotifers fed only within the biofilm (Table 3; see discussion). Ingestion rates and grazing pressure of the biofilm-dwelling rotifer community on cyanobacteria (particularly on filamentous species, Table 3) were high compared with those exerted on diatoms and on the total phototrophic biomass.

**Table 3:** Impact of the rotifer community grazing on the biofilm total phototrophic community.

	**Ingestion rate (µg pigment cm^-²^ day^-1^**)			**Grazing pressure (% day^-1^**)		
	**Nov-08**	**Apr-09**	**Mar-10**	**Nov-08**	**Apr-09**	**Mar-10**
Microphytobenthos (Chl *a*-eq)	0.2-0.7	0.6-1.8	0.3-0.9	1.4-4.3	1.4-4.4	0.3-1.1
Cyanobacteria (Myxoxanthophyll + zeaxanthin)	0.01-0.04	0.14-0.45	0.04-0.12	0.2-0.7	3.9-12.2	3-9.4
Filamentous cyanobacteria (Myxoxanthophyll)	0.01-0.03	0.12-0.36	0.03-0.1	0.3-1.1	5-16	9-28
Diatoms (Chlc+fucoxanthin+diadinoxanthin)	0.03-0.11	0.04-0.13	0.08-0.25	0.07-0.22	0.02-0.06	0.01-0.04

Range of mean daily ingestion rates and grazing pressure of the rotifer community on the biomass of total phototrophic community (in terms of Chl *a*-eq), cyanobacteria, filamentous cyanobacteria and diatoms (in terms of biomarker pigments), in the biofilm (n=3).

**Figure 3:**
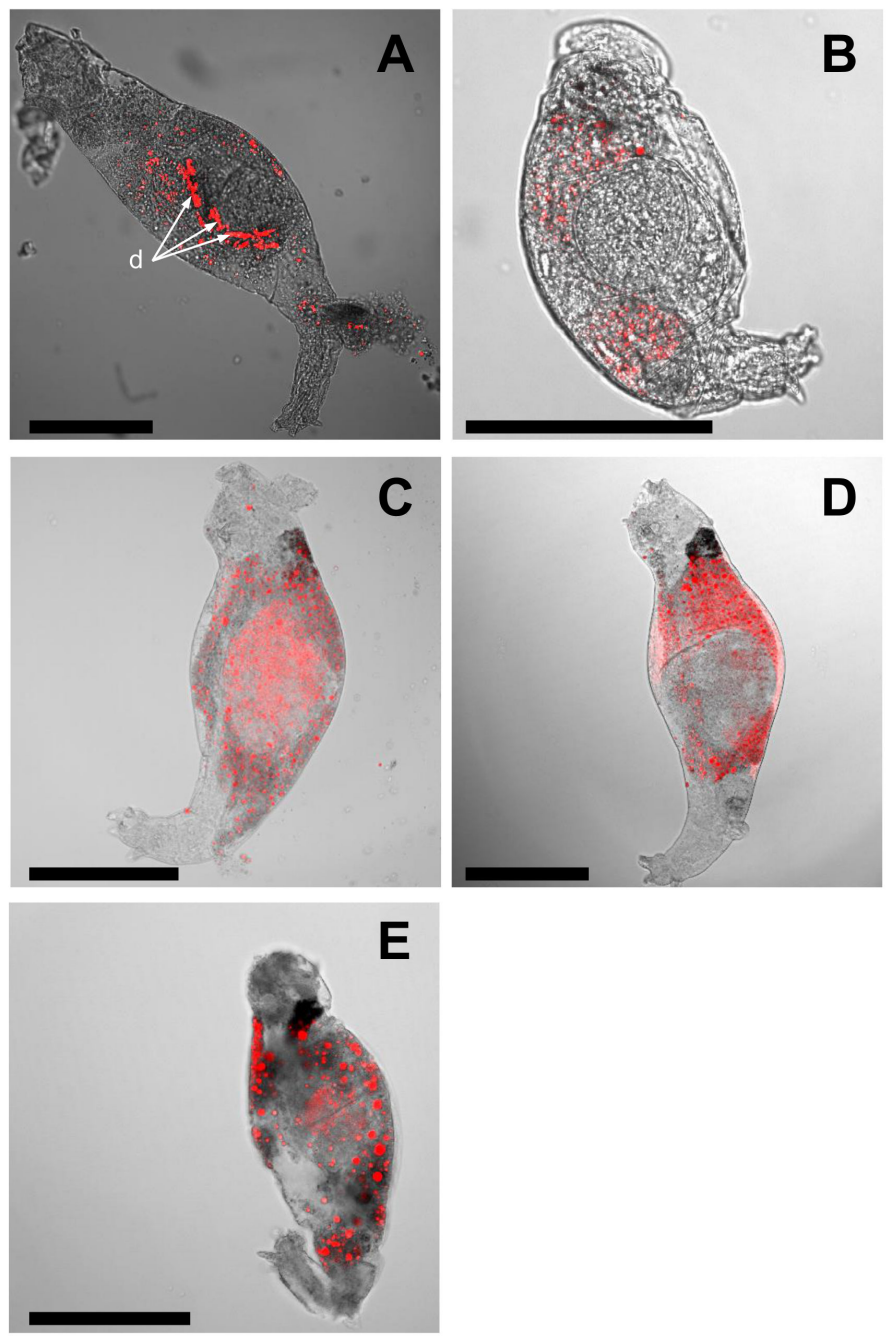
Chlorophyll fluorescence (red objects) in five bdelloid rotifer individuals under a confocal laser scanning microscope. Scale bar is 100 µm. d: ingested diatoms. (A–E) Five randomly selected bdelloid individuals.

Between 67–317 fluorescent objects (FO) were detected by CLSM in centremost layers of five bdelloid rotifer individuals (Figure 3A–E). Mean FO area was 14 µm^2^ spanning 1.8–416 µm^2^. Mean and median FO circularity were high: 0.8 and 0.9, respectively—a perfect circle has a circularity of 1. FO distribution from rotifer boundaries was normal (*N* = 692; χ^2^ = 89, *Df* = 16, *P* < 0.001). The area and fluorescence (CTCF) of FO correlated positively with increasing distance from rotifer boundaries (*N* = 692; area: *R* = 0.11, *P* < 0.01; CTCF: *R* = 0.1, *P* < 0.01). The circularity of FO correlated negatively with increasing distance from rotifer boundaries (*N* = 692; *R* = –0.14, *P* < 0.001). These trends are echoed by the observation of large body-centred fluorescent rods (i.e. ingested diatom cells: *d* on Figure 3A). Small round FO were more evenly distributed in rotifer bodies.

## Discussion

It must be envisaged that the presence of myxoxanthophyll in rotifer bodies could result from symbiotic associations between cyanobacteria and the invertebrates. To the best of our knowledge, observations of endosymbiotic cyanobacteria have not been reported yet for rotifers. However, it is known that bdelloids have foreign genes deriving from prokaryotes, parasitic fungi, plants, algae, and protists, by horizontal gene transfer (HGT) [44]. HGT processes in rotifers are still not well understood. Among the hypotheses, it is proposed that the source organisms of HGT could be either ingested prey or endosymbionts [45]. Nevertheless, endosymbiotic cyanobacteria were observed in neotropical snails forming rounded corpuscles in the midgut gland [39] and in temperate sponges [40]. These symbiotic cyanobacteria belong to Chroococcale, Pleurocapsale and Synechococcale orders, including non-filamentous species. However, myxoxantophyll pigment is the signature of filamentous cyanobacteria [42] and the fluorescent objects (FO) observed in bdelloids in our study were all circular (Figure 3), suggesting that myxoxanthophyll in rotifers, did not originate from endosymbiotic cyanobacteria. We also observed a large variation of FO area per individual (up to 231-fold) weakening the hypothesis that FO could be symbiotic cells. A more plausible rationale for FO is that bdelloids possess digestive circular vesicles which can include partly digested material [46]. Within bdelloids, we observed that fringing FO were small and showed the lowest fluorescence. This is consistent with the hypothesis that bdelloids contained digestive vesicles—including partly digested algal material—originating from ingested cyanobacteria and migrating towards rotifer body fringes during digestion.

The maximum feeding pressure exerted by bdelloid rotifers on the total MPB biomass in the biofilm was low, as it was previously observed for nematodes in the same site of the Garonne river [18]. Values for rotifer ingestion rates were however on average 70 fold higher than those of nematodes, resulting in a 14 fold higher grazing pressure exerted on the MPB biomass by rotifers than by nematodes.

It is generally known that meiofauna feed on diatoms [11,18,29,31,47], and the fact that biofilm-dwelling rotifers live in diatom-rich biotopes makes it logic that they use these algae as food [6] as observed in this study. However, as our results showed that the fucoxanthin/Chl *a*-eq ratio was lower in the animals than in the feeding medium, diatoms were clearly not selected by the bdelloids, on the contrary, they were probably avoided. Previous studies have highlighted that many species of diatoms can produce secondary metabolites (oxylipins) with toxic effects on marine invertebrates such as planktonic copepods (e.g. *Calanushelgolandicus* ) and cladocerans for example 48 but to the best of our knowledge, no data on diatom toxicity on bdelloid rotifers is available. Thus, further investigation is needed to examine the cause of the observed limited ingestion of diatoms: toxic effect or a morphological effect (e.g. size of food particles [26]). Green microalgal biomarker pigments (lutein and chlorophyll b) were not detected in rotifers indicating that they were not used as food source by the rotifers. It can be alternatively envisaged that both diatom and cyanobacterial pigments could have been either strongly degraded during their passage through rotifer guts. However, although chlorophyll b is a labile pigment, lutein and fucoxanthin in contrast, has been often found in pigment gut contents of meiofauna indicating that they are particularly resistant to degradation [29,31,47].

Unexpectedly, our results showed that the relative cyanobacterial biomass (represented by the myxoxantophyll /Chl*a*-eq ratio) in rotifer body was much higher than the proportion of filamentous cyanobacterial biomass in the biofilm [42]. These results show that the rotifers were able to selectively ingest benthic filamentous cyanobacteria from the biofilm, and additionally, that rotifers can exert a high feeding pressure on these filamentous cyanobacteria in comparison with the pressure exerted on diatoms (Table 3). Assuming a relatively low microphytobenthic production of the biofilm at a daily scale (a turnover of 8 and 16 days for grazed and ungrazed stream epilithic biofilms respectively, according to Lamberti & Resh [49]), this grazing activity suggests that rotifer community could regulate microphytobenthic cyanobacteria stocks by selective feeding. Previous studies hypothesized that direct top-down control of MPB biomass by meiofaunal grazing activity is not a primary regulating mechanism. Meiofauna play a secondary role by modifying the potential bottom up controls of MPB through, e.g. bioturbation [6,11]. While observations previously reported for nematodes support this hypothesis [18], our results however, suggest that for rotifers, in contrast, this question merits further investigations. The possibility that biofilm rotifers also fed partially on phytoplankton in the water column should also be kept in mind [16], especially as the Garonne’s high flow rates provide a constant replenishment of phytoplankton passing over the biofilms [50]. However, considering the low Chl*a* concentration measured in the Garonne river at the study site [50], it seems unlikely that the biofilm rotifers obtained a considerable fraction of their food from the water column.

*Cyanobacteria* as a preferred prey for rotifers is surprising since: 1) diatoms dominated both biofilm MPB, while filamentous cyanobacteria were minor contributors to biofilm MPB biomass and 2) cyanobacteria are generally considered as non-attractive preys because they show poor nutritional quality (low EPA and P contents [51,52]) together with chemical and structural defence against grazing [51-53]. Some marine harpacticoid copepods have been reported to avoid ingesting cyanobacteria in marine superficial sediments [29,31,47]. However, it is known that *Brachionus* planktonic rotifers are able to ingest some cyanobacteria but their growth and their toxin sensitivity are variable according to the cyanobacterial species fed upon [53,54]. Battaglia & Valencia [55] mention that in an Antarctic lake, HPLC analyses of pigments in the bdelloid rotifer *Philodina* *gregaria* Murray 1910, revealed accumulations of myxoxanthophyll and other carotenoids that are characteristic of the cyanobacterial mat on which they feed. However, no rotifer pigment content data were provided by these authors. Gaudes et al. [56] report that the biomass of *Diplogaster* *rivalis* (Schultze, 1857), the most common meiobenthic nematode species in cyanobacterial biofilms of the Llobregat river (Spain) was positively correlated to the availability of cyanobacterial biomass, suggesting that these nematodes could consume cyanobacteria. These authors hypothesised that cyanobacterial high protein content could compensate the lack of polyunsaturated fatty acids in grazer diet.

In rivers, benthic rotifers can be highly selective during food collection - size of preys being the most important discriminating factor for selection [26]. The main cyanobacteria present in the studied biofilm are the filamentous taxa *Phormidium* *uncinatum* (Gomont, 1890), *Lyngbia* sp., and *Phormidium* sp. [57]. They are mat forming cyanobacteria with long unbranched filaments (1–5 × 10–50 µm) organized in fine and smooth layered strata [58]. Unfortunately, knowledge about bdelloid rotifer food-size preferenda is lacking. Nevertheless, even if the biomass contribution of cyanobacteria is minor, filamentous cyanobacterial cells may provide a higher “encounter rate” to biofilm-grazers than diatom cells.

Cyanobacteria produce and release a number of volatile organic compounds (VOC) which may be infochemicals and are responsible for musty odours of water [59,60]. This is the case for the cyanobacterial species present in the studied biofilm: *Phormidium* *uncinatum* *,* *Phormidium* sp. and *Lyngbia* sp. produce geosmin, dihydroβ-ionone, β-ionone and 2-methylisoborneol [58,59]. Dihydroβ-ionone is an attractant for the nematode *Caenorhabditis elegans* (Maupas, 1900), and this cyanobacterial chemotaxis effect differs among nematode species [59]. Chemotaxis of nematodes may affect both their capacity to locate nearby biofilms and to position themselves within a biofilm [61,62]. Some planktonic rotifers are able of chemical recognition of substances (e.g. genus *Brachionus*) [61,62]. It is plausible that chemotaxis aids biofilm-dwelling rotifers to detect cyanobacteria within the biofilm. Some studies highlight the ingestion of chemically defended plant tissues by invertebrates as a strategy to deter predators. In laboratory, *Hyalella* *azteca* (Saussure, 1858), a freshwater amphipod was less preyed upon when its diet was based on a chemically defended macrophyte, *Berula* *erecta* (Coville, 1893) [63]. Some laboratory experiments showed that small crustaceans can develop a toxin resistance, mostly after a period of pre-exposition [64]. As some cyanobacteria are well known for their toxic properties (e.g. [65] for *Phormidium* *species* ), their hypothetical ingestion by benthic rotifers as a strategy to reduce predation deserves further exploration.

Echinenone was detected in the rotifers, but not in the biofilm nor in the phytoplankton samples. Thus, the echinenone detected within bdelloids was not gained through cyanobacteria ingestion. Echinenone is a β-carotene derivative pigment that has been found in many invertebrates (e.g. in molluscs, crustaceans, echinoderms and tunicates [43,66,67]). Nevertheless, bdelloids possess carotenoid biosynthetic genes, originating from other organisms by horizontal gene transfer [44]. It seems that these genes allow production of carotene pigments and derivatives by rotifers [68]. Thus, since β-carotene was also a major pigment found in rotifers and in microphytes (Table 1, Figure 1 a, b), it can be hypothesised that rotifers were able to convert β-carotene to echinenone. Gilchrist & Green have shown that the two major groups of rotifers (Bdelloidea and Ploima) can accumulate carotenoids from their algal food and convert these into other carotenoid pigments (e.g. astaxanthin and astacene) [69]. However, authors did not observe accumulation of echinenone but of astaxanthin and astacene in the Bdelloidea rotifer *Philodina* *roseola* (Ehrenberg, 1832), likely because they used a chromatography method which did not allow separating the carotenoids contained in rotifer samples prior to their identification. Considering that astaxanthin and echinenone have a similar spectral shape but different retention times, it is possible that these authors were unable to distinguish the presence of echinenone in their rotifer samples. It has been previously hypothesized that the photoprotection of microalgae, assured by the accumulation of photoprotector carotenoids in their cells, could be transferred to grazers in the form of pigments. In addition, the survival of certain species of meiobenthic copepods is directly related to their carotenoid content when exposed to copper [70]. In this context, our results suggest that the potential link between the accumulation of echinenone and a possible improvement of resistance to metal toxicity and UV light exposures deserves to be tested for bdelloid rotifers.

## Conclusion

To conclude, our results showed that biofilm-dwelling rotifers fed on cyanobacteria and diatoms. It is strongly suggested that this feeding was highly selective on cyanobacteria while diatoms were avoided. Our results support the hypothesis that the role of cyanobacteria as food source for the meiobenthos has been underestimated as previously suggested [59]. We feel that both the potential of biofilm-dwelling rotifers to control cyanobacterial development and the idea that cyanobacteria are ingested by the rotifers to obtain predation protecting toxins merit further investigation. Another aspect soliciting further interest is the apparent potential of bdelloid rotifers to convert microphyte-ingested β-carotene to the photoprotective pigment echinenone.

## Supporting Information

Figure S1 **Epilithic biofilm in the Garonne River.**
(TIF)
