# Supplementary figures and images for: Selective Feeding of Bdelloid Rotifers in River Biofilms
Source: PLoS One. 2013 Sep 20;8(9):e75352. doi: 10.1371/journal.pone.0075352 (PMC3779155; doi:10.1371/journal.pone.0075352)

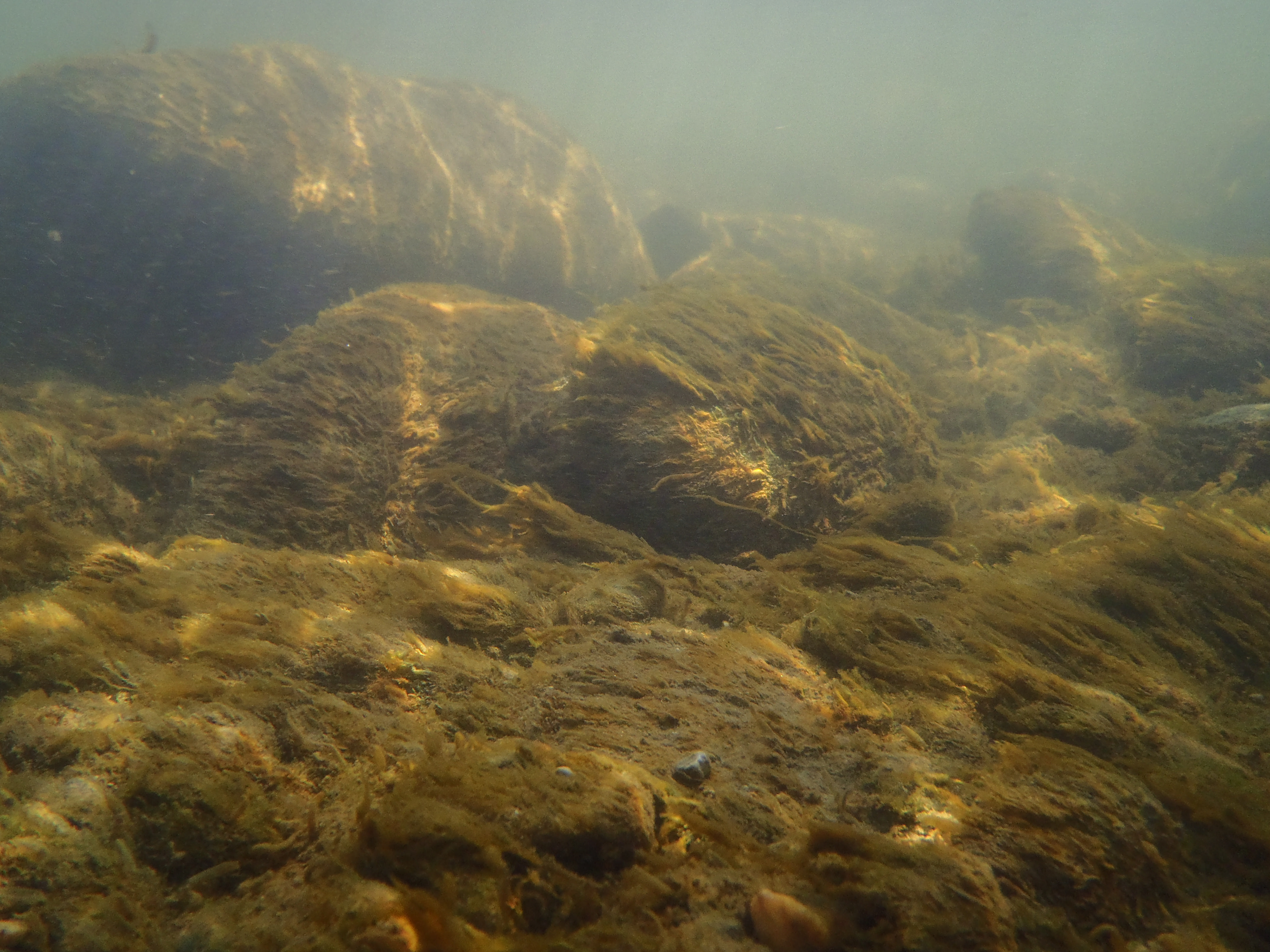

Supplement: Figure S1 — Epilithic biofilm in the Garonne River. (TIF) [file pone.0075352.s001.tif]
